# Perianal Abscess in a Patient With Central Diabetes Insipidus: A Case Report

**DOI:** 10.7759/cureus.88235

**Published:** 2025-07-18

**Authors:** Yuxin Du, Zongqi He

**Affiliations:** 1 Department of Anorectal Surgery, Kunshan Hospital Affiliated to Nanjing University of Chinese Medicine, Kunshan, CHN

**Keywords:** case report, central diabetes insipidus, hypernatremia, perianal abscess, perioperative management

## Abstract

Central diabetes insipidus (CDI) is a rare endocrine disorder that can pose significant risks in the perioperative setting, especially when routine fasting and fluid restriction are applied. We report the case of a 34-year-old man with a history of CDI who underwent surgical drainage of a perianal abscess and subsequently developed severe hypernatremia and neurological symptoms due to postoperative water restriction. Prompt fluid resuscitation and endocrinology consultation led to rapid recovery. This case underscores the importance of early recognition and individualized perioperative management, including desmopressin replacement and tailored fluid strategies in CDI patients undergoing surgery.

## Introduction

Perianal abscess is a common infectious condition that requires prompt surgical intervention [1]. Central diabetes insipidus (CDI) is a rare disorder characterized by the excretion of large volumes of dilute urine. Clinically, it typically presents with intense thirst (polydipsia) and excessive urination (polyuria) [2]. The pathophysiological basis of CDI lies in impaired synthesis or transport of arginine vasopressin (AVP) in the hypothalamic supraoptic or paraventricular nuclei. This defect leads to reduced water reabsorption in the renal collecting ducts, resulting in the loss of large amounts of dilute urine. The consequent increase in plasma osmolality stimulates the thirst center in the hypothalamus, leading to excessive thirst and compensatory water intake [2,3]. Therefore, patients with CDI who have no history of cranial trauma, surgery, or underlying disease and who can drink water normally typically do not develop hypernatremia.

CDI significantly alters the patient’s ability to retain water, and during the perioperative period, routine fasting and fluid restriction may result in critical electrolyte disturbances. Reports of surgical management in CDI patients are scarce.

We present a case of a patient with CDI who developed acute postoperative hypernatremia following drainage of a perianal abscess. The case highlights the importance of individualized care for patients with underlying endocrine disorders.

## Case presentation

A 34-year-old man was admitted to the Department of Anorectal Surgery, Kunshan Hospital of Traditional Chinese Medicine, Kunshan, China, on May 17, 2025, with a 10-day history of perianal swelling and pain. Physical examination revealed a tender, erythematous, fluctuant mass at the 1 o’clock position in the perianal area, consistent with a perianal abscess.

He had a known history of CDI but was not on desmopressin therapy at the time of admission. Preoperative laboratory evaluation showed normal serum electrolytes: potassium 4.19 mmol/L, sodium 142.1 mmol/L, chloride 107.3 mmol/L, calcium 2.47 mmol/L (Table 1, Time A). Urinalysis revealed a urine specific gravity of 1.005 (normal reference range: 1.010-1.025) (Table 1, Time B). No other significant abnormalities were found in the remaining laboratory tests. Routine preoperative fasting and water restriction for 6 hours were applied. On May 19, 2025, the patient underwent successful drainage under spinal anesthesia. According to the anesthesiologist’s recommendation, the patient underwent routine fasting and water restriction for 6 hours postoperatively. Later that day at 16:45, he developed muscle twitching, altered consciousness, and aphasia. When the patient developed altered mental status and aphasia, vital signs, including blood pressure, heart rate, and blood oxygen saturation, remained within normal ranges, as monitored by ECG. The point-of-care blood glucose level was 5.3 mmol/L. The patient was relatively young and had no history of psychiatric illness or other underlying diseases, apart from a known diagnosis of CDI. Given this background, we promptly performed laboratory tests, including serum electrolytes and renal function, and urgently contacted the endocrinology team for consultation. After bedside evaluation, the endocrinologist considered that the neurological symptoms were likely caused by dehydration due to preoperative fasting and fluid restriction in the context of CDI. Based on this clinical judgment, stroke was not considered, and a head CT was not performed at that time. Emergency laboratory testing revealed severe hypernatremia: sodium 178.0 mmol/L, chloride 140.5 mmol/L, potassium 4.19 mmol/L, calcium 2.65 mmol/L (Table 1, Time C).

**Table 1:** Laboratory test results Time A: electrolyte test result at 13:55 on May 17, 2025; Time B: urine-specific gravity test result at 09:22 on May 18, 2025; Time C: electrolyte test result at 18:09 on May 19, 2025; Time D: electrolyte test result at 23:08 on May 19, 2025.

Time	Test Item	Result	Reference Range	Unit
A	Sodium	142.1	137–147	mmol/L
	Chloride	107.3	99–110	mmol/L
	Potassium	4.19	3.5–5.3	mmol/L
	Calcium	2.47	2.11–2.52	mmol/L
B	Urine-specific gravity	1.005 ↓	1.01–1.025	–
C	Sodium	178.0 ↑	137–145	mmol/L
	Chloride	140.5 ↑	98–107	mmol/L
	Potassium	4.19	3.5–5.1	mmol/L
	Calcium	2.65 ↑	2.1–2.55	mmol/L
D	Sodium	158.7 ↑	137–145	mmol/L
	Chloride	123.2 ↑	98–107	mmol/L
	Potassium	3.84	3.5–5.1	mmol/L
	Calcium	2.48	2.1–2.55	mmol/L

The patient was given 500 mL of 5% glucose intravenously, and the patient was also advised to drink water freely. His symptoms improved significantly. Repeat electrolytes at 23:08 showed sodium 158.7 mmol/L, chloride 123.2 mmol/L, potassium 3.84 mmol/L, and calcium 2.48 mmol/L (Table 1, Time D). By the following morning, electrolytes had normalized, and the patient was asymptomatic.

An endocrinology consultation was formally conducted during the hospitalization to assist in managing the hypernatremia. After the sodium reached 158.7 mmol/L, serial sodium levels were monitored daily for four consecutive days. He made an uneventful recovery and was discharged on postoperative day 6 with instructions to resume endocrinology follow-up. At the time of discharge, the patient’s serum sodium level was 147 mmol/L. The patient was advised to follow up with endocrinology after discharge. The patient continued to manage CDI through free water intake and was not prescribed desmopressin. No antibiotics were prescribed upon discharge, as the patient had already undergone incision and drainage.

## Discussion

CDI is a rare but potentially life-threatening condition caused by a deficiency in AVP, resulting in hypotonic polyuria and compensatory polydipsia [4,5]. Its perioperative management poses distinct challenges that are often under-recognized in surgical settings, especially for non-endocrine specialists [6].

Our case highlights the peril of applying standard fasting protocols in patients with CDI, leading to a rapid onset of symptomatic hypernatremia following routine surgery. The combination of perioperative water deprivation, the absence of desmopressin therapy, and surgical stress likely precipitated this electrolyte crisis.

Recent literature has underscored the risks associated with poor inpatient awareness and management of CDI. A large international survey of more than 1,000 patients with CDI found that 13% of hospitalized patients did not receive desmopressin during fasting states, leading to significant dehydration, and that 26% experienced hyponatremia requiring hospitalization due to desmopressin misuse [5]. In our case, the opposite occurred - lack of desmopressin with concurrent fluid restriction resulted in hypernatremia.

Furthermore, the survey revealed that 64% of patients with CDI reported impaired quality of life and 80% encountered health-care professionals who confused CDI with diabetes mellitus [5]. This emphasizes the need for better clinical education and protocol development [5,7,8]. Our case adds to this concern, demonstrating how a lack of endocrine consultation in perioperative planning could rapidly lead to complications.

From a physiological perspective, the absence of vasopressin prevents renal water reabsorption in the collecting ducts, resulting in massive urinary losses. During periods of fluid restriction, such as pre- and post-surgical fasting, patients are unable to compensate for insensible losses, leading to progressive hypernatremia. Tomkins et al. emphasized that even minor surgical stress can disrupt the fragile water balance in CDI patients and recommend that desmopressin be considered an essential medication during the perioperative period to be administered even during fasting states [3].

Our case reinforces several clinical imperatives: (1) routine preoperative fasting protocols should be modified in CDI patients, (2) close perioperative electrolyte monitoring is mandatory, even in minor surgeries, (3) endocrinology teams should be involved early, ideally preoperatively, and (4) desmopressin must be administered during fasting states if fluid intake is restricted.

## Conclusions

While CDI remains rare, it is increasingly recognized that it is not benign, and mismanagement can result in significant morbidity. This case adds to the growing call for standardized inpatient protocols to ensure continuity of care for patients with rare endocrine disorders. Patients with CDI require careful perioperative planning to prevent severe electrolyte disturbances such as hypernatremia. This case demonstrates the importance of early recognition, fluid management, and multidisciplinary coordination. Individualized protocols should be considered in patients with rare endocrine comorbidities undergoing surgery.
